# Pathogenicity and virulence of *Ralstonia solanacearum* species complex: Understanding its virulence mechanisms for proper management

**DOI:** 10.1080/21505594.2026.2721851

**Published:** 2026-08-20

**Authors:** Masayuki Tsuzuki, Yasufumi Hikichi, Sora Tateda, Akinori Kiba, Kouhei Ohnishi

**Affiliations:** Faculty of Agriculture and Marine Science, Kochi University, Nankok, Japan

**Keywords:** Ralstonia pseudosolanacearum, phytopathogenic bacteria, infection process, virulence, quorum sensing

## Abstract

The soil-borne Gram-negative beta-proteobacterium *Ralstonia solanacearum* species complex (RSSC) causes bacterial wilt, a devastating plant disease that threatens crop production and food security worldwide. In this review, we first summarize current knowledge of RSSC pathogenicity and virulence, focusing on the factors that determine its ability to infect and cause disease, including advances in resistance breeding and the genetic basis of host resistance to bacterial wilt. Next, we highlight the questions provided from the previous studies and describe our recent studies, which revealed that the *phc* QS network is a highly complex regulatory system that dominates global gene expression and finely tunes RSSC virulence throughout the infection process, from root epidermis invasion to colonization of xylem vessels. Finally, we identify key knowledge gaps and discuss future research directions and practical strategies for the effective management of bacterial wilt.

## Introduction

Bacterial wilt is a devastating crop disease caused by the soil-borne Gram-negative beta-proteobacteria *Ralstonia solanacearum* species complex (RSSC). RSSC causes substantial economic losses and threatens global food security, making RSSC one of the most important phytopathogenic bacteria worldwide. Indeed, RSSC has been ranked as the second most important phytopathogenic bacterium based on its scientific and economic impact [1]. Like many plant pathogens, individual RSSC strains exhibit host specificity; however, the species complex as a whole infects a remarkably wide range of plants. The most important hosts are solanaceous crops, including eggplant, tomato, tobacco, and potato [2]. RSSC also infects monocot crops such as ginger and banana. Disease spread is strongly influenced by agricultural practices, including inoculation of soil from infected roots, dissemination by farm implements such as through the pruning of tomato plants, and, in the case of banana Moko disease, insect vectors [3].

Based on phylogenetic analyses and phenotypic characteristics, RSSC is currently classified into three species and four phylotypes: *Ralstonia solanacearum* (phylotypes IIA and IIB), *R. pseudosolanacearum* (phylotypes I and III), and *R. syzygii* (phylotype IV) [4–6]. Despite their genetic diversity, RSSC strains share many biological and pathogenic traits, and phylotype classification broadly reflects differences in host range and ecological characteristics [2].

A key determinant of RSSC virulence is the *phc* quorum-sensing (QS) system, which regulates gene expression in response to cell population density [7]. In this review, we first describe the general features of RSSC pathogenicity and virulence. We also review progress in resistance breeding and the genetic basis of host resistance to bacterial wilt. We then summarize recent studies revealing how multiple two-component regulatory systems interact with *phc* QS, forming a far more complex regulatory network than previously recognized. We also examine evidence from microscopic studies indicating that *phc* QS contributes to the fine-tuning of virulence throughout the infection process, from root epidermis invasion to xylem vessel colonization. Finally, we highlight key unanswered questions and discuss future directions for the effective management of bacterial wilt.

## General features of RSSC pathogenicity and virulence

### Infection process

RSSC primarily invades host plants through root wounds or sites of secondary root emergence, while insect-mediated aerial transmission has been reported for certain banana-infecting strains [8]. Microscopic studies of infected tomato roots revealed that the pathogenic strain GMI1485 enters through the extremities and axils of secondary roots and subsequently colonizes the intercellular spaces of the root cortex and vascular parenchyma [9]. Following invasion, the bacteria degrade plant cell walls and spread through the vascular system.

RSSC synthesizes and secretes a range of plant cell wall-degrading enzymes (PCWDEs) via the type II secretion system, including endoglucanase (Egl) [10], endopolygalacturonases (PglA, PehB, and PehC) [11,12], pectin methylesterase (Pme) [13], and β-1,4-cellobiohydrolase (CbhA) [14]. These enzymes degrade the cell walls of cortical cells and xylem vessels, promoting the spread of bacteria. Although the contribution of each individual enzyme to virulence is limited, genetic studies suggest that their combined activity is essential for full virulence [14].

### Interactions between RSSC and host plants involved in host immunity

Disease development by RSSC results from complex interactions between the pathogen and its host. Successful colonization requires the bacteria to overcome host pattern-triggered immunity (PTI) [15–17]. As in many plant and animal pathogenic bacteria, RSSC pathogenicity relies heavily on the bacterial type III secretion system (T3SS) and its transported type III effectors (T3Es) to suppress host defenses and promote infection [18].

### Type III secretion system (T3SS)

The type III secretion system (T3SS) is a major virulence determinant of RSSC. T3SS comprises multiple component proteins and delivers effector proteins, termed type III effectors (T3Es), from the bacterial cells directly into host cells, where they suppress innate immune responses and promote infection and induction of symptoms. The T3SS in RSSC is encoded by the *hrp* gene cluster, a megaplasmid-borne region containing more than 20 genes encoding both T3SS structural components and regulatory proteins involved in *hrp* gene expression via signaling cascades [19–22].

Genetic studies have revealed the importance of the T3SS for RSSC pathogenicity. Eight avirulent Tn5-insertion mutants were found to carry mutations within the *hrp* gene cluster [21,23]. Notably, an *hrcV* mutant of strain GMI1000, which encodes a T3SS component, retained the ability to invade tomato roots but exhibited greatly reduced multiplication within the host, indicating that successful infection requires both root invasion and T3SS-mediated suppression of host defenses [24–26].

RSSC possesses an unusually large repertoire of T3Es. More than 110 T3Es have been identified among over 140 RSSC strains using sequence-based searches, comparative genomics, functional analyses, genetic screening, and T3E-translocation analyses [27]. Individual strains encode 45–76 T3Es, with an average of 64 T3Es, substantially more than reported for other phytopathogenic bacteria such as *Pseudomonas syringae* (average 31) and *Xanthomonas campestris* (average 23) [27–29]

**Figure 1.**
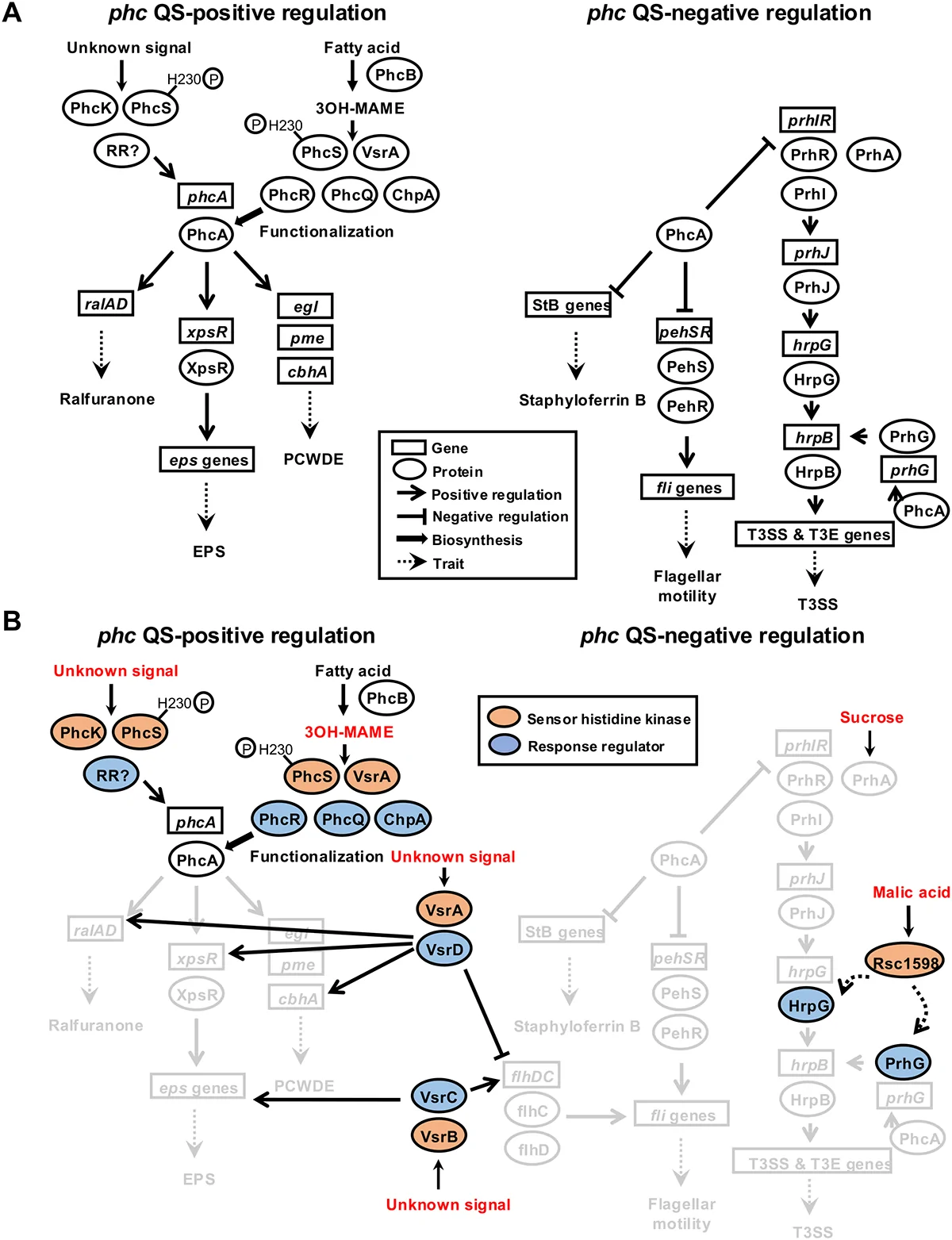
Signaling pathways around *phc* QS system in RSSC: (A and B) the pictures representing signaling pathways related to *phc* QS. (A) Signaling pathways with the corresponding traits positively or negatively regulated by *phc* QS. RR, response regulator. (B) Another version of signaling pathway depiction. The contribution of two-component system, sensor histidine kinase and response regulator, and corresponding input signaling compounds are emphasized by coloring. The signaling pathways are represented based on Genin and Denny (2012) and later studies.

Expression of *hrp*/*hrc* genes and T3E *rip* genes is controlled by HrpB, an arabinose operon regulatory protein-type (AraC-type) transcriptional regulator [30] (Figure 1(A)). HrpB harbors a 25-base *cis*-regulatory element, termed the plant-inducible promoter (PIP) box, and is itself regulated by environmental signals. The *hrp* gene cluster and the large number of T3E genes are activated dependent on HrpB and the PIP-box sequence. Expression of *hrpB* and its downstream targets is induced when the bacteria are cultured with plant cell suspensions or in minimal media, suggesting responsiveness to both plant-derived and metabolic/nutrient signals [19,31]. Plant sugars such as sucrose, glucose, and fructose are sensed by the outer membrane TonB-dependent receptor PrhA, which activates a downstream signaling cascade, PrhR-PrhI-PrhJ-HrpG-HrpB [32,33] (Figure 1(A)). HrpG, an OmpR family response regulator, is essential for *hrpB* expression and pathogenicity [34]. Malic acid also induces the *hrp* regulon through the *RSc1598*-encoding histidine kinase, which likely activates HrpG via phosphorylation [33] (Figure 1(B)). In addition, transcriptome studies revealed that HrpG regulates pathways beyond the T3SS [35]. Notably, chemotaxis toward malic acid mediated by the methyl-accepting chemotaxis protein McpM promotes bacterial movement toward tomato roots [36].

Following suppression of pattern-triggered immunity and successful host colonization, bacterial populations increase and activate *phc* quorum sensing (QS), a process whereby bacteria secrete and sense QS signals. The QS signals accumulate in the extracellular space, leading to the regulation of bacterial gene expression in response to fluctuations in cell population density. In the *phc* QS-active state, the LysR-type transcriptional regulator PhcA suppresses *prhIR* expression, thereby inhibiting T3SS and T3E deployment [37,38] (Figure 1(A)). RSSC also possesses PrhG, a paralog of HrpG with high sequence similarity [39]. Although PrhG is not essential for pathogenicity, it is involved in the up-regulation of HrpB, similar to HrpG, especially under nutrient-limited conditions and during the QS-active state [39–41] (Figure 1(A)).

### Type III effectors (T3Es)

Type III effectors (T3Es) promote RSSC pathogenicity by suppressing host innate immunity and manipulating plant cellular processes to facilitate infection and symptom development [42]. Super-multiple deletion analysis revealed extensive functional redundancy among T3Es: deletion of at least 42 T3Es abolished virulence on infiltrated tobacco leaves, although weak virulence remained following petiole inoculation [43]. Individual T3Es contribute to pathogenicity through diverse mechanisms, including suppression of plant basal defense responses, modulation of host plant metabolism, and interference with the recognition of other effectors. Comprehensive reviews of T3E functions have been conducted by Macho [44], Landry et al. [27], and Hu et al. [45].

In some host–pathogen combinations, T3Es are recognized by plant immune receptors and trigger host defense responses, often accompanied by a hypersensitive response (HR), an extreme defense response of host plants, resulting in incompatibility between the pathogen and host. For example, RipAA and RipP1 (formerly AvrA and PopP1, respectively) induce HR in *Nicotiana* species [45–49]. Additional examples include the recognition of RipJ in *Solanum pimpinellifolium* LA2093, RipAZ1 in *S. americanum*, AWR5 and RipTPS in *Nicotiana* species, RipBS and RipS6 in potato, and RipAX2 in *S. torvum* [45,50–54].

The corresponding immune receptors have been identified in some cases; for example, RipP2 (formerly PopP2) is recognized in *Arabidopsis* by the paired immune receptors RRS1-R and RPS4 [55–59]. In *Nicotiana benthamiana*, RipY is detected by the plasma membrane coiled-coil (CC) nucleotide-binding leucine-rich repeat receptor (CNL) RRS-Y, which activates immune responses [60]. A resistance locus from the wild tomato *S. lycopersicoides* LA4245 encodes a CNL protein that recognizes RipBN and confers resistance [61]. Similarly, RipB functions as an avirulence factor, as RipB-mediated resistance is lost in *Roq1*-silenced *N. benthamiana* plants [62].

### Other bacteria-derived factors

In addition to T3Es, several RSSC-derived molecules contribute to host–pathogen interactions by functioning as both virulence factors and immune elicitors. Exopolysaccharide (EPS), a major virulence factor of RSSC, is recognized by tomato immune receptors and triggers host plant defense responses [63,64]. The non-RD receptor kinase SlLYK4, previously identified as a chitin receptor and is a fungus-derived elicitor inducing host defense responses [65], mediates EPS perception in *R. solanacearum* and contributes to resistance against bacterial wilt [66]. SlLYK4 forms a receptor complex with the RD receptor kinases SlLYK1 and SlLYK13, both of which are also required for host resistance. Given the importance of EPS in RSSC virulence, its recognition represents an effective host plant defense strategy.

Other pathogen-derived molecules also activate plant immunity. PehC, an exo-polygalacturonase secreted by RSSC, promotes the release of plant pectin-derived oligogalacturonic acids. These molecules act as damage-associated molecular patterns (DAMPs), inducing DAMP-triggered immunity (DTI) in tomato and other solanaceous species [67].

Xylem-specific immunity may involve the lectin-receptor-like kinase LORE, which activates the 2C protein phosphatase LOPP and is thought to function through reversible phosphorylation-dependent signaling [68,69]. Several bacterial molecules, including medium-chain 3-hydroxy fatty acids, rhamnolipid precursors, and lipopolysaccharides, have been proposed as LORE-associated microbe-associated molecular patterns, although the mechanisms of their recognition remain unclear [70,71].

### Plant-derived factors

Recent studies have identified plant cell wall composition as a host factor that contributes to RSSC infection. The resistant tomato cultivar “Hawaii 7996” responds to *R. solanacearum* infection by inducing the formation of a vascular structural barrier composed of ligno-suberin coating and tyramine-derived hydroxycinnamic acid amides. Overexpression of genes involved in the ligno-suberin pathway enhances resistance to *R. solanacearum* in susceptible tomato cultivars [72]. FERONIA (FER) negatively regulates immunity against *R. solanacearum* by suppressing lignin biosynthesis and promoting degradation of the NAC transcription factor RD26, a positive regulator of resistance [73,74]. In contrast, cellulose synthases (CESAs), which are involved in cell wall biosynthesis, contribute to resistance against *R. solanacearum* [75,76]. Mutations in the secondary cell wall genes *IRX5*/*CESA4*, *IRX3*/*CESA7*, or *IRX1*/*CESA8* increase resistance against *R. solanacearum* [76]. In contrast, the *Arabidopsis* mutant *ixr1*/*cev*, which carries a partial loss-of-function mutation in CELLULOSE SYNTHASE 3 (CESA3), a vital enzyme required for primary cell wall synthesis, exhibits a similar wild-type susceptibility [76]. However, a genome-wide association (GWA) identifies a resistance-associated quantitative trait locus (QTL) at the *CESA3* locus [75,77], and the *cesa3*^*mre1*^ mutant shows altered primary cell wall structure and enhanced resistance to *R. solanacearum* [75].

Mutations in *IRX5*/*CESA4*, *IRX3*/*CESA7*, or *IRX1*/*CESA8* are associated with up-regulation of abscisic acid-responsive (ABA-responsive) defense genes, while ABA mutants (*abi1-1*, *abi2-1*, and *aba1-6*) show increased susceptibility [76]. In contrast, *cesa3*^*mre1*^ constitutively expresses the jasmonic acid-responsive genes *PDF1.2* and *VSP1* [7,5]. These findings suggest that alterations in both primary and secondary cell wall biosynthesis enhance resistance against *R. solanacearum* by modulating plant hormone-mediated defense pathways.

RSSC targets these defense mechanisms through its effectors. RipAC promotes pathogenicity by disrupting the interaction between the CSI1 and CESA proteins, reducing cellulose content and altering root development [78]. Although CSI1 also interacts with FER, RipAC specifically affects the CESA–CSI1 complex without disrupting CSI1–FER interactions, thereby promoting infection while avoiding immune activation. Another effector, RipU from *R. solanacearum* K60 (RipU^K60^), a contributor to bacterial virulence, alters actin and microtubule organization, which may affect secondary cell wall formation in xylem tissues and facilitate vascular colonization [79]. These findings indicate that cell wall structure is both a key component of host defense and a major target of RSSC virulence strategies.

Additional resistance-associated factors have been identified through activity-based proteome analysis. Infection of the tolerant tomato cultivar “Hawaii 7996” specifically activates papain-like cysteine proteases and serine hydrolases in the leaf apoplast [80]. Among these proteins, the subtilase P69D contributes to resistance against *R. solanacearum* in tomato [81]. Proteomic analyses of xylem sap and apoplastic fluids further revealed the accumulation of PR1 proteins during infection. Surprisingly, loss of PR1b enhances resistance, likely through compensatory up-regulation of homologous genes [82]. The PR1 proteins are processed by an unknown protease into CAPE peptides, which restrict *R. solanacearum* growth by reprogramming defense gene expression, highlighting another layer of host immunity against bacterial wilt.

### Virulence mechanisms

Virulence reflects the ability of a pathogen to cause disease in its host. Because virulence factors must be deployed at specific stages and locations during infection, virulence mechanisms are closely linked to the infection route [83].

### *Initial model of* phc *QS*

The role of *phc* QS in virulence was first elucidated in studies of *R. solanacearum* strain AW1. In the proposed model, the LysR-type transcriptional regulator PhcA is activated in response to the fatty acid-derived QS signal 3-hydroxypalmitate (3-OH PAME), which is synthesized by the methyltransferase PhcB. The signal is sensed by the sensor histidine kinase, PhcS, which transmits a phosphorylation signal to the response regulator PhcR. In the absence of 3OH-PAME, PhcR represses PhcA activity to regulate target gene expression [84–87]. However, this model could not fully explain *phc* QS regulation because some RSSC strains did not respond to 3OH-PAME, and mutations in PhcS or PhcR did not completely abolish *phc* QS-activated traits. Our biochemical studies resolved the first question by identifying an alternative QS signal, 3-hydroxymyristate (3-OH MAME), which is used by some strains instead of 3OH-PAME [88]. Recent findings about second question are described in “The recent progress addressing some questions provided by the previous studies”.

### Phc *QS-regulated virulence factors*

In the *phc* QS-active state, production of virulence factors is largely controlled by the global regulator PhcA [89]. PhcA regulates multiple traits by activating genes involved in virulence factor production while repressing genes associated with motility and T3SS/T3E production [32] (Figure 1(A)). Transcriptomic analyses have shown that PhcA controls the expression of hundreds of genes through both positive and negative regulation [90,91]. Consequently, genes positively regulated by PhcA are up-regulated, whereas genes negatively regulated by PhcA are down-regulated in the *phc* QS-active state.

As noted above, EPS is a major virulence factor in RSSC [92]. EPS I, the predominant extracellular polysaccharide, plays a critical role in wilt symptom development [93,94], and an EPS I-deficient mutant shows severely impaired stem colonization [95]. Recent work showed that EPS I confers biofilm mobility, enabling passive colony expansion in response to mechanical deformation [96]. These findings suggest that EPS I enhances the viscoelastic properties of RSSC biofilms, thereby promoting bacterial dissemination in xylem vessels.

EPS I production is induced by *phc* QS through XpsR, a major regulator of the *eps* operon whose expression is activated by PhcA [97]. The two-component systems VsrAD and VsrBC provide an additional regulatory layer within the QS network [87] (Figure 1(B)). The histidine kinase VsrA and response regulator VsrD, together with PhcA, are required for induction of *xpsR* expression [97,98]. In contrast, the histidine kinase VsrB and the response regulator VsrC activate the *eps* operon in cooperation with XpsR, although the signals sensed by these systems remain unknown [99]. Another transcriptional regulator, EpsR, also contributes to *eps* operon activation; however, its overexpression suppresses EPS I production, suggesting a concentration-dependent regulatory role [100,101].

Valls et al. [35] revealed that HrpG, a key regulator in the HrpB cascade, up-regulates certain virulence factor-related genes, including the lectin-encoding genes (*lecM* and *lecF*) and the PCWDE-encoding genes (*pehB* and *egl*). Because these virulence factor-related genes are also targets of the *phc* QS system, and because the HrpB cascade is suppressed in the *phc* QS-active state [37,38], RSSC appears to employ a multilayered regulatory network that maintains the expression of virulence factor-related genes across diverse *in planta* environments.

Strain-specific virulence has also been investigated in the pandemic RSSC phylotype II (*R. solanacearum*) race 3 biover 2 (R3bv2) lineage, which causes potato brown rot [102]. Compared with the cold-sensitive tropical strain GMI1000, the R3bv2 strain UW551 showed enhanced survival in potato tubers at 4°C and increased virulence at 20°C [103–105]. In addition, strain UW551 can partially overcome the resistance of the tomato cultivar “Hawaii 7997” by detoxifying inhibitory compounds in xylem sap [106]. However, low-temperature virulence is not restricted to R3bv2 strains, and some R3bv2 strains display only moderate virulence under cool conditions. These observations suggest that low-temperature virulence is a quantitative trait governed by complex genetic and regulatory mechanisms rather than a characteristic unique to the R3bv2 lineage [107].

### Bacterial wilt-resistant cultivars

The development and deployment of resistant cultivars is an effective strategy for managing bacterial wilt. Studies of host resistance to RSSC have not only supported resistance breeding programs but have also provided genetic insights into the determinants of RSSC virulence and pathogenicity. In tomato, quantitative trait loci (QTLs) associated with bacterial wilt resistance have been identified at multiple genomic regions, including several on chromosome 6 [108–115]. Notably, chromosome 6 also contains the *Mi* locus, which confers resistance to both bacterial wilt and root-knot nematodes [116].

The QTLs associated with tomato resistance to RSSC appear to vary depending on the pathogen strain. For example, resistance in the tomato cultivar “Hawaii 7996” is largely governed by strain-specific QTLs against certain RSSC isolates [106,113,115]. Furthermore, combining QTL analysis with imaging-based quantification of disease progression has enabled the identification of resistance loci detected by conventional visual assessments as well as additional loci that can be detected before symptoms become apparent to human observers [117].

Recently, genome-wide association (GWA) studies in *Arabidopsis* [77,118–120], tomato [121,122], and potato [123] have provided novel insights into the genetic basis of host resistance to RSSC. In *Arabidopsis*, a GWA study conducted under different temperature conditions identified the involvement of a strictosidine synthase-like 4 (SSL4) protein in the early response to *R. pseudosolanacearum* strain GMI1000 [77]. Another study, which quantified *Arabidopsis* root growth following GMI1000 inoculation, revealed the involvement of host cytokinin signaling in pathogen-induced root growth inhibition [118]. In addition, a GWA study identified BACTERIAL WILT SUSCEPTIBILITY 1 (BWS1) as a susceptibility factor for *R. solanacearum* in *Arabidopsis*. BWS1 directly interacts with SUPPRESSOR OF G2 ALLELE OF skp1, whereas the interaction was suppressed by the T3E RipAC [120]. Together, these findings demonstrate the utility of GWA approaches for identifying genes and pathways involved in host resistance and susceptibility to bacterial wilt.

Strain-specific resistance remains an important challenge for bacterial wilt management. An imaging-based QTL analysis using an *R. solanacearum* strain identified novel QTLs that were not detected in previous QTL analyses using *R. pseudosolanacearum* strains, highlighting the contribution of strain-specific resistance mechanisms [11,7]. Therefore, resistance breeding programs should consider the correspondence between host resistance QTLs and the diversity of RSSC strains. Expanding QTL analyses to include a wider range of host varieties and pathogen strains should improve our understanding of resistance mechanisms and increase the likelihood of developing cultivars with durable and broad-spectrum resistance.

QTL pyramiding, the breeding strategy of introducing multiple resistance QTLs into host genomes, is a promising approach for improving resistance to bacterial wilt [124]. However, the remarkable diversity and abundance of T3Es in RSSC may pose a challenge to this strategy. Because many T3Es exhibit functional redundancy, the loss or modification of a single effector can enable RSSC strains to evade effector-triggered resistance while retaining virulence functions through alternative effectors [27]. Consequently, the durability and effectiveness of pyramided resistance should be carefully evaluated under field conditions before practical deployment.

## The recent progress addressing some questions provided by the previous studies

**Figure 2.**
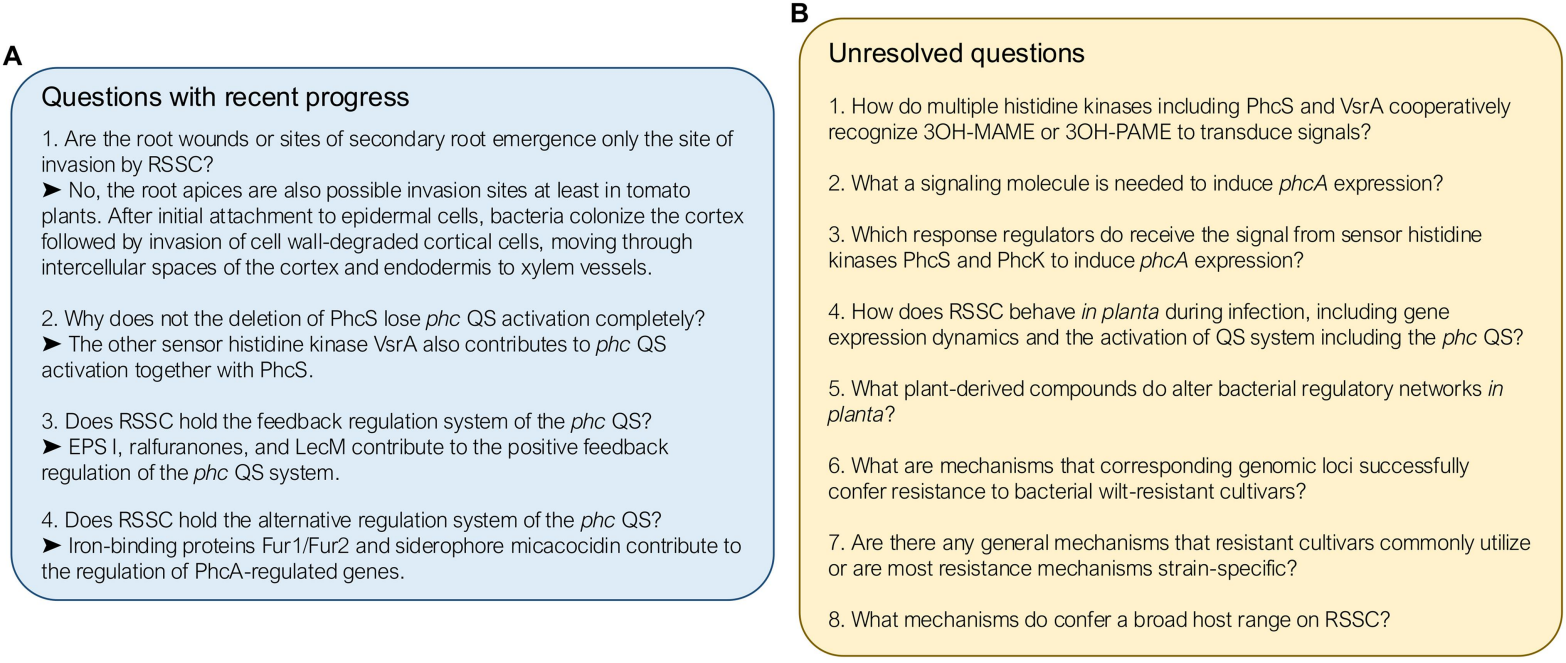
Lists of questions about RSSC pathogenicity and virulence. (A) A list of questions with the recent findings. (B) A list of unresolved questions mentioned in this review.

In this section, we describe the recent progress addressing some questions provided by the previous studies (Figure 2(A)).

### Are the root wounds or sites of secondary root emergence only the site of invasion by RSSC?

**Figure 3.**
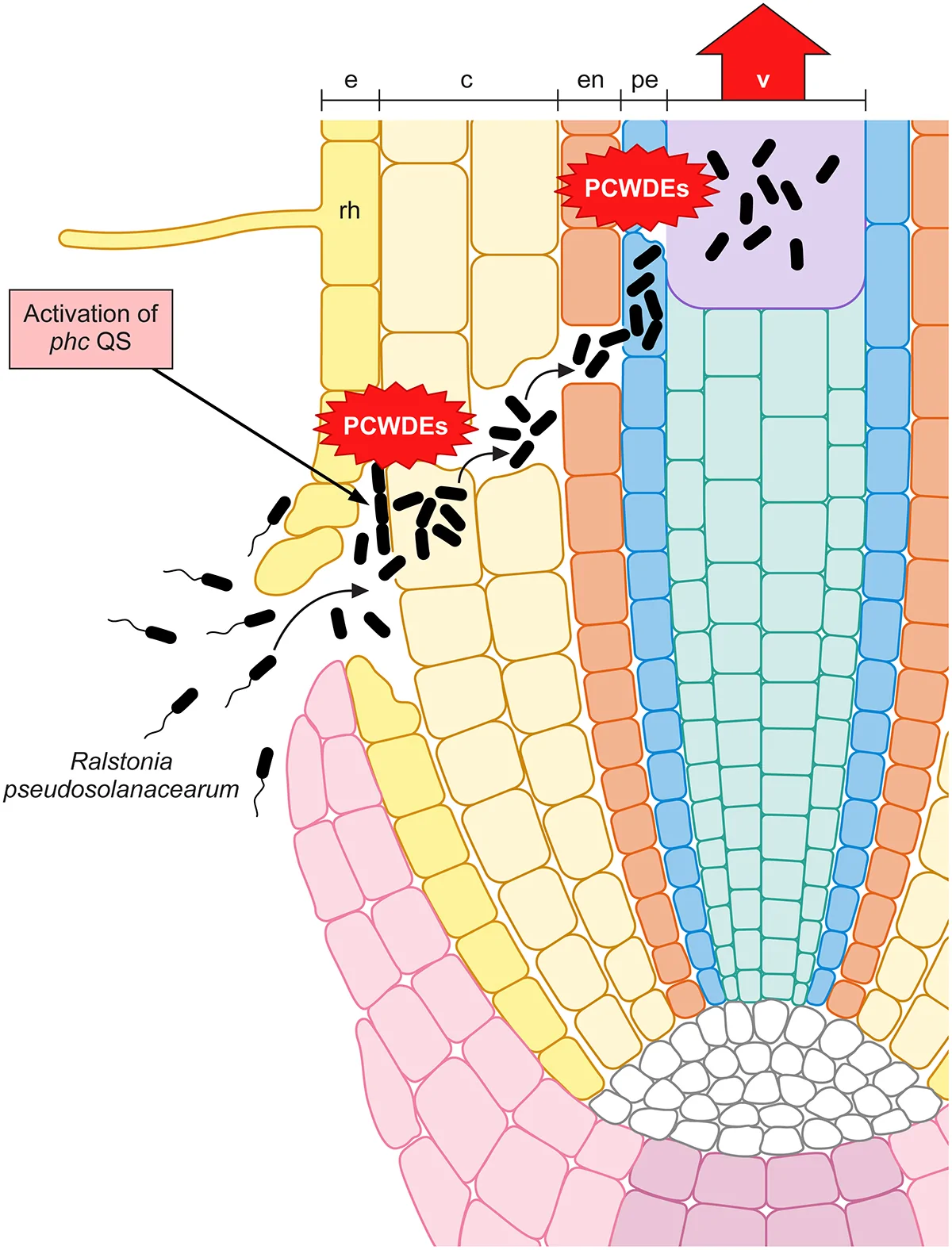
Model of infection route and alteration in behaviors of *Ralstonia pseudosolanacearum* strain OE1-1 through root apexes: the picture showing the tentative infection route and position-dependent alteration of behaviors of*Ralstonia pseudosolanacearum* strain OE1-1 through root apexes with indication of tentative places where*phc*QS is activated: root hair; e, epidermal cell; c, cortical cell; en, endodermis; pe, pericycle cell; v, xylem vessel, PCWDEs, plant cell wall degradation enzymes.

As described above, it was thought that RSSC primarily invades host plants through root wounds or sites of secondary root emergence. Our recent study examined the infection process in greater detail using four-day-old tomato seedlings lacking secondary roots and co-incubated with the RSSC phylotype I strain, *R. pseudosolanacearum* strain OE1-1. Microscopic observations were performed to investigate the infection route through the tomato root apices [125,126]. The bacteria initially attach to epidermal cells in the root elongation zone, and subsequently colonize the cortex followed by invasion of cell wall-degraded cortical cells with a denatured nucleus adjacent to the epidermis (Figure 3). In the cortical cells, OE1-1 forms characteristic mushroom-shaped biofilms that are thought to promote bacterial dissemination through intercellular spaces of the cortex and endodermis, enabling infection of pericycle cells and xylem vessels. Ultimately, extensive xylem colonization leads to wilt symptom development on the tomato plants. The successive changes in bacterial behavior observed during infection suggest that RSSC can sense *in planta* environmental signals and precisely regulate virulence during different stages of colonization.

### Why does not the deletion of PhcS lose QS activation completely?

This question was subsequently addressed through omics studies using strain OE1-1, which produces 3-OH MAME as the QS signal, revealing the involvement of novel two-component system proteins and complex regulatory mechanisms. The sensor histidine kinase, PhcS, is thought to perceive 3-OH MAME [7], whereas another sensor histidine kinase, PhcK, helps maintain *phcA* expression and supports PhcA-dependent gene regulation [127] (Figure 1(A)). Although disruption of *phcS* alone does not impair QS-dependent phenotypes, simultaneous deletion of PhcS and the sensor histidine kinase VsrA abolishes these phenotypes, suggesting that PhcS and VsrA cooperatively mediate 3-OH MAME perception [128] (Figure 1(A)). Likewise, the H230Q amino acid substitution in PhcS, which eliminates autophosphorylation, results in loss of QS-dependent phenotypes, reduced *phcA* expression, and loss of virulence on tomato, indicating that PhcS contributes both to *phc* QS signal transduction and maintenance of *phcA* expression [128,129]. The response regulator genes PhcR and PhcQ are located downstream of *phcS* [86]. Mutation of PhcR partially impairs *phc* QS-dependent phenotypes, whereas deletion of PhcQ abolishes *phc* QS-dependent phenotypes and virulence on tomato [130]. In addition, the response regulator ChpA contributes to multiple traits, including virulence, and most *phc* QS-dependent traits, except swimming motility [131–133]. Together, these findings suggest that PhcR, PhcQ, and ChpA independently modulate PhcA-mediated gene regulation (Figure 1(A,B)).

Quorum quenching, which disrupts QS signaling, is a promising strategy for controlling bacterial virulence through the use of QS-inhibitory compounds. Because quorum quenching targets virulence rather than bacterial growth, it may provide a sustainable alternative to antibiotic-based disease management [134]. The *phc* QS inhibitors (PQIs) have been identified and shown to attenuate *phc* QS-dependent traits and virulence in strain OE1-1, highlighting their potential for bacterial wilt control [135]. Virulence-targeting management strategies offer the potential for sustainable and durable disease control because they impose less risk of the emergence of resistant bacterial strains.

### *Does RSSC hold the feedback regulation system of the* phc *QS?*

QS is tightly connected with the production of various types of chemical compounds, QS signals. Acyl homoserine lactones are a major class of QS signal used by Gram-negative proteobacteria for intraspecies QS [136]. LuxI and LuxR are essential for the QS control of bioluminescence in *Vibrio fischeri*. LuxI is the synthase in the QS signal *N*-(3-oxohexanoyl)-L-homoserine lactone. LuxR is the cytoplasmic receptor for the QS signal, as well as the transcriptional activator of *luxICDABE* operon. When the QS signal accumulates, it is bound by LuxR, and the LuxR–acyl homoserine lactone complex recognizes a consensus binding sequence upstream of the lux operon, activating its expression. Because *luxI* expression is activated by the QS signal-bound LuxR, QS signal production is induced. On the other hand, we had no information on the feedback regulation of the *phc* QS. Notably, our transcriptome analysis recently showed that EPS I is involved in a positive feedback loop within the *phc* QS system, raising the possibility that it functions as an intercellular signaling mediator that modulates the expression of PhcA-regulated genes during *phc* QS activation [137].

Biosynthesis of the aryl-furanone secondary metabolites, ralfuranones, depends on *ralA* and *ralD*, whose expression is induced by PhcA in the *phc* QS-active state [138,139]. Deletion of *ralA* reduces virulence on tomato, indicating that ralfuranones contribute to virulence [138]. Ralfuranones also play a role in the formation of mushroom-shaped biofilms in strain OE1-1 [140]. Notably, both the *ralA* deletion mutant and the EPS I-deficient mutant (∆*epsB*) exhibit gene expression profiles similar to that of ∆*phcA*, suggesting that ralfuranones and EPS I contribute to the maintenance of *phc* QS activation through a positive feedback mechanism [141]. Recently, the TetR/AcrA family transcriptional regulator RalT, whose production is dependent on ralfuranones but independent of *phc* QS, has been reported to participate in ralfuranone-mediated positive feedback regulation of the *phc* QS system [142].

Lectins are carbohydrate-binding proteins encoded by three genes in RSSC, namely, *lecM*, *lecF*, and *lecX* [143]. In strain OE1-1, LecM shows affinities to mannose, fructose, fucose, galactose, and arabinose [144]. In the *phc* QS-inactive state, *lecM* expression is positively regulated by HrpG, and LecM contributes to bacterial attachment to plant surfaces. Upon *phc* QS activation, *lecM* is positively regulated by PhcA. Notably, the *lecM* mutant in the *phc* QS-active state exhibits a transcriptomic profile similar to that of ∆*phcA* and shows reduced extracellular stability of 3-OH MAME, suggesting that LecM participates in a positive feedback loop that maintains *phc* QS activation [145]. These findings indicate that LecM functions as a virulence factor in strain OE1-1. In addition, LecF and LecX are fucose- and xylose-binding lectins, respectively. A double-deletion mutant lacking both lectins displays enhanced attachment under static conditions but reduced attachment under fluid conditions, suggesting that the multiple lectins contribute to adaptation to changing extracellular environments during host infection [143].

During colonization of the root cortex, strain OE1-1 activates the *phc* QS system [125] and induces production of CbhA, which is required for invasion of the cortical cells [146]. Transcriptome analyses further revealed that deletion of *cbhA* significantly reduces *phcA* expression and alters the expression of PhcA-regulated genes and QS-dependent phenotypes, similar to the effects of *phcA* deletion. Together, these results suggest that CbhA is involved in the full expression of *phcA*, thereby contributing to the QS feedback loop.

### *Does RSSC hold the alternative regulation system of the* phc *QS?*

Iron uptake is essential for bacterial survival because iron serves as a co-factor for numerous redox enzymes and iron-binding proteins [147]. Recently, we showed that two ferric uptake regulator (Fur) proteins, Fur1 and Fur2, cooperatively regulate gene expression, including PhcA-regulated genes, under iron-rich conditions, thereby controlling iron uptake, bacterial growth, and virulence in strain OE1-1 [148]. To acquire ferric iron, strain OE1-1 produces the phenolate-type polycarboxylate siderophore staphyloferrin B and the phenolate-type pyochelin siderophore micacocidin, which function as major iron-scavenging molecules. Analysis of micacocidin-deficient mutants revealed that micacocidin contributes to the regulation of PhcA-regulated genes responsible for virulence as well as ferric iron-scavenging activity [149].

## Unresolved questions and future directions

In this section, we summarize unresolved questions about RSSC pathogenicity and virulence, listed in Figure 2(B), and discuss future directions. As described above, it has been revealed that *phc* QS is regulated by multiple two-component system proteins. However, molecular mechanisms by which multiple two-component system proteins regulate the *phc* QS regulatory network remains unclear. The recent insights remind us of other questions: How do multiple histidine kinases including PhcS and VsrA cooperatively recognize 3OH-MAME or 3OH-PAME to transduce signals? Furthermore, what a signaling molecule is needed to induce *phcA* expression, and which response regulators do receive the signal from PhcS and PhcK to induce *phcA* expression (Figure 1(A,B); 2B-1, −2, and −3)? To advance the discovery and rational design of anti-virulence compounds, a deeper understanding of the *phc* QS regulatory network is required. In particular, the mechanisms by which QS signals are perceived and subsequently transduced through multiple two-component system proteins remain largely unresolved. Elucidating the roles of each individual sensor histidine kinase and its associated signal transduction pathways can advance our understanding of RSSC virulence regulation and support the development and optimization of virulence-targeted control strategies, including quorum quenching.

Understanding how RSSC behaves *in planta* during infection, including its gene expression dynamics and the activation of the *phc* QS system, is essential for the development of effective disease management strategies (Figure 2(B–4)). Imaging analyses of both RSSC and host plants are required for elucidating the mechanistic links between bacterial gene expression and the infection process. Transcriptome analyses of bacteria conducted under diverse environmental conditions have provided valuable insights into global gene expression dynamics throughout the bacterial life cycle [150,151]. However, because bacterial populations often exhibit cell-to-cell heterogeneity in gene expression, the application of single-cell approaches is likely to be important for understanding bacterial behavior at both the individual-cell and population levels [152].

*In vivo* transcriptome analyses of bacterial cells in plant stems have revealed how plant-derived signals influence bacterial gene expression patterns [90,153]. Extending such analyses to additional stages of RSSC infection can provide a more comprehensive understanding of how RSSC responds to host cues and modulates its behavior during disease development. In particular, identifying the plant-derived compounds that alter bacterial regulatory networks *in planta* and elucidating their mechanisms of action are crucial for understanding RSSC virulence and pathogenicity (Figure 1(B); 2B-5). Such knowledge may contribute to the development of novel and more effective strategies for bacterial wilt control.

Further investigation of resistance QTLs identified in RSSC-resistant varieties is also needed (Figure 2(B–6)). Although the introduction of resistance QTLs into cultivars is an important breeding objective, identifying the causal polymorphisms within these QTLs and elucidating their resistance mechanisms are equally important for advancing our understanding of bacterial wilt resistance. Recent studies have highlighted the importance of strain-specific resistance [117], suggesting that the accumulation of genomic sequences and QTL data from diverse host plant varieties should support the development of more effective resistance‑breeding strategies and reveal how general the mechanisms resistant cultivars commonly utilize are or whether most resistance mechanisms are strain-specific (Figure 2(B–7)). In addition, the current collection of sequenced RSSC genomes remains insufficient to fully characterize the evolutionary dynamics of this species complex. Expanding the number and diversity of available genome sequences should improve our understanding of the genetic determinants of the broad geographic distribution, host range, and strain-specific pathogenicity of RSSC. Such knowledge should contribute to both the development of durable resistance and the refinement of disease‑management strategies.

Elucidating the factors driving the broad host range and global distribution of RSSC remains a major challenge (Figure 2(B–8)). Experimental evolution studies have shown that adaptive mutations in EfpR, a global catabolic repressor in RSSC, can arise during *in planta* adaptation and enhance bacterial fitness in alternative host species [154]. Similarly, genes involved in the *phc* QS regulatory system have been identified as targets of adaptive mutations during long-term exposure to acidic conditions and during legume symbiosis [155,156]. These findings suggest that, in addition to the extensive repertoire of type III effectors (T3Es), adaptive changes in *efpR* and *phc* QS-related genes may play important roles in host adaptation and environmental versatility. Greater availability of genomic resources may enable the identification of the genetic mechanisms responsible. The development of additional experimental tools and resources is also crucial for advancing bacterial wilt research. Compared with model species such as *Arabidopsis* and the legume *Lotus japonicus*, genetic and genomic resources remain relatively limited for many economically important solanaceous hosts.

In summary, continued efforts to elucidate the mechanisms governing RSSC virulence and pathogenicity are critical for the development of effective and sustainable strategies for crop protection against bacterial wilt. Successful implementation of these strategies requires the parallel advancement of both fundamental knowledge of bacterial wilt pathogenesis and its translation into practical approaches compatible with agricultural production systems.

## Data Availability

There is no data associated with this research.
